# Effects of the Space Holder Shape on the Pore Structure and Mechanical Properties of Porous Cu with a Wide Porosity Range

**DOI:** 10.3390/ma17123008

**Published:** 2024-06-19

**Authors:** Jian Xiao, Yanping He, Wenjun Ma, Yiheng Yue, Guibao Qiu

**Affiliations:** 1 School of Material Science and Engineering, Jiangxi University of Science and Technology, Ganzhou 341000, China; hyp1234562023@163.com (Y.H.); 15359783512@163.com (W.M.); 19142171189@163.com (Y.Y.); 2 College of Materials Science & Engineering, Chongqing University, Chongqing 400044, China

**Keywords:** porous metal, metal foam, copper, powder metallurgy, compression performance

## Abstract

Porous copper (Cu), with varying porosities, has been made using carbamide as a space holder through the powder metallurgy route. Two shapes of carbamide particles were used, (i) needlelike and (ii) spherical, in order to investigate the effect of the space holder shape on the pore structure and mechanical properties of porous Cu. The compressive deformation behavior of porous Cu was studied under a compression test. The pores’ structural characteristics and mechanical properties of the porous Cu varied significantly with the shape of the space holder. Although the effect of the space holder shape on the porosity was not regular, the effect on the mechanical properties was regular. The stress increased monotonically with the increase in the strain, and strain hardening occurred at the plastic yield stage. The elastic modulus and yield strength followed the power law, with the relative density irrespective of the space holder shape. The empirical constants associated with different empirically developed power law relations were different, according to the shape of space holder. A quantitative relationship between the elastic modulus and yield strength and the spacer content was obtained to control the mechanical properties of the present porous Cu or other porous metals and metal foams using the well-known space holder method.

## 1. Introduction

Porous copper (Cu) has been widely used for heat dissipation, as a battery motor and a catalyst carrier, as well as for other applications [1]. The use of space-holding filler materials is an indirect method to fabricate porous Cu [2]. In this method, the Cu powder is either filled into a “dry” bulk of fillers, a suitable solvent, or even an organic binder to mix the space holders and the metal powders.

For porous Cu based on the space holder method, the earliest literature can be traced back to 2005 [3]. The paper described a lost carbonate sintering process for manufacturing open cell metal foams. Cu foams with a porosity of 50–85% and cell sizes of 53–1500 μm were manufactured. Since then, the use of this method to prepare porous Cu with a foam structure has become increasingly popular, and the space holder is still in use today. In the past few decades, a large number of studies have focused on the effect of the content and particle size of the space holder on the pore structure and mechanical properties of porous Cu. According to these studies, the porosity of the porous Cu increased with the increase in the space holder content, while the mechanical properties decreased. For example, Shirjang et al. [4] stated that the porosity of copper foams increased from 50.2 to 71.8%, and the plateau stress decreased from 83 ± 3.1 MPa to 20 ± 1.8 MPa, as the content of K_2_CO_3_ used as the space holder increased from 50% to 70%. The porosity of the porous copper increased from 38.48 ± 0.91% to 66.47 ± 1.06%, and the yield strength decreased from 42.8 ± 4.6 MPa to 6.9 ± 0.9 MPa when the space holder of bimodal K_2_CO_3_: NaCl was 1:2, as their contents increased from 30% to 70%, according to Ray et al. [5] The total porosity of porous copper increased from 27.5% to 67.1%, and the elastic modulus (C_11_) decreased from 59.4 ± 0.2 GPa to 1.0 ± 0.2 GPa when the content of K_2_CO_3_ used as the space holder increased from 0 to 54.4%, according to Jana et al. [6]. Luo et al. [7] showed that the elastic modulus of titanium foam decreased with the increasing size of the magnesium particles, while the porosity decreased, etc. However, little attention was paid to the shape of the space holder, even when the study of the shape of the metal powder extended the scope to the entire porous metal. For example, Jain et al. [8] studied the effects of the particle shape on the microstructure and compressive response of 316L SS foam using the space holder technique. Mondal et al. [9] studied the effect of the particle shape and strain rate on the microstructure and compressive deformation response of pure Ti-foam made using acrowax as the space holder. The existing studies showed that the particle size of the space holder had little influence on the pore structure and mechanical properties of porous Cu compared with its content. However, it is not clear whether the influence of the shape of space holder on the pore structure and mechanical properties of porous Cu is negligible compared with the content.

Therefore, the effect of the space holder shape relative to its content on the pore structure and mechanical properties of porous Cu was studied in this work. Based on the author’s previous research [10,11,12], spherical carbamide with two sizes was selected as the space holder due to its high quality, low price, and easy removal at low temperature.

## 2. Materials and Methods

**Figure 1:**
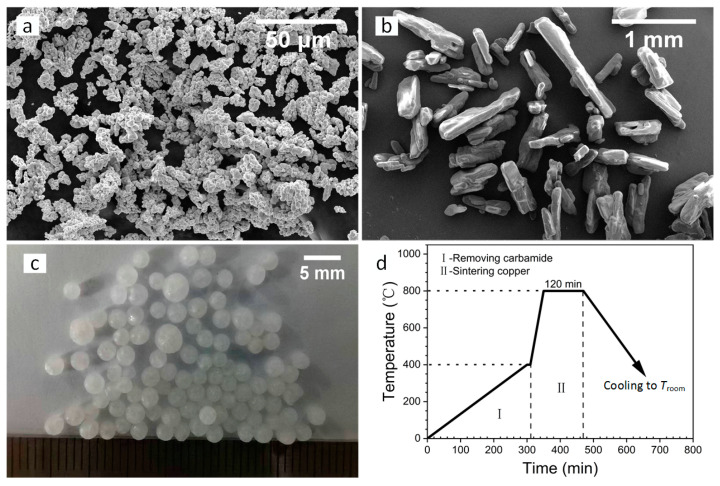
Images of the powder and space holder: (**a**) Cu and (**b**) needlelike and (**c**) spherical carbamide. (**d**) The one step of heat treatment for the fabrication process of the porous Cu.

The raw material of the metal powder was selected as Cu powder (purity: ~99.9%, size: ≤50 μm) with a coralloid shape (see Figure 1a), using an electrolytic process. The Cu powder was purchased from Beijing Hongyu New Material Technology Co., Ltd. (Beijing, China). The carbamide with two shapes, needlelike and spherical, was used as the space holder, as shown in Figure 1b,c. The carbamide was purchased from Xilong Scientific Co., Ltd. (Chengdu, China) and sieved with 40–80 mesh for the needlelike particles, while the spherical carbamide was 0.85–3 mm. The diameter and height of the sample were designed as 20 mm and 8 mm, respectively. The volume content of the space holder was set as 10, 20, 30, 40, 50, 60, 70, and 80 vol.%, respectively. According to the density of the Cu powder (8.96 g/cm^3^) and carbamide (1.335 g/cm^3^), the weight of the Cu powder and carbamide particles were calculated. The weights of the metal powder and needlelike space holder particles were increased by 0.05 g, considering their mass loss. For spherical carbamide, it was formulated according to the calculated value due to its large particle size.

We poured the measured Cu powder and carbamide into the mortar, added an appropriate amount of anhydrous ethanol, and stirred. After mixing evenly, it was put into the steel mold for pressing and forming, and the pressing pressure and holding time were 200 MPa and 0.5 min, respectively. A layer metal mesh support in a porcelain boat was used to place the obtained green compacts. One step of heat treatment (see Figure 1d was used to determine the compacts in a vacuum tube furnace. The temperature increased from room temperature (*T*_room_) to 400 °C within 300 min, and the temperature was held for 15 min to remove the carbamide particles in part-I; then, the temperature continued to increase to 850 °C within 45 min, and the temperature was held for 2 h to sinter the Cu powder in part-II. After that, the furnace cooled to room temperature to obtain the porous Cu samples.

The mass volume method was used to calculate the porosity of the porous Cu samples, and their porosity in each spacer content was considered as the average value of ten samples to ensure repeatability and statistical data. The internal structure and cell wall morphology of the porous Cu samples were observed using a scanning electron microscope (FEI Company, Hillsboro, OR, USA). An electronic universal testing machine (Sunstest, Shenzhen, China) was carried out to measure the compression performance of the porous Cu samples with a 2 mm/min of head movement rate at room temperature. The mechanical properties of porous Cu were given as the average value of five samples.

## 3. Results and Discussion

### 3.1. Pore Structure

**Figure 2:**
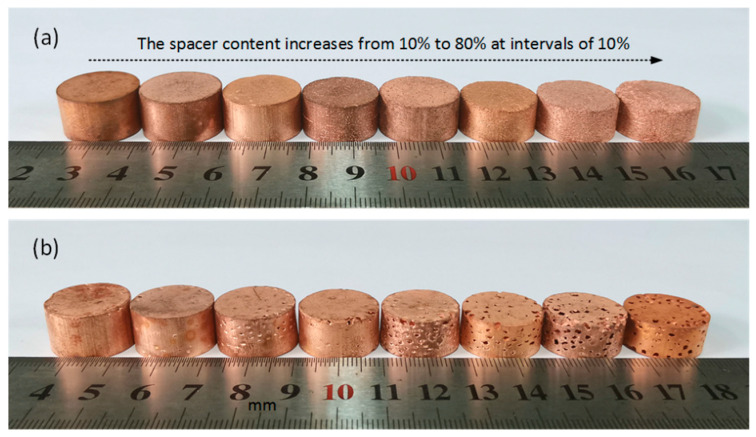
Digital images of the porous Cu samples fabricated using different shapes of carbamide as the space holder with a volume content between 10 and 80%: (**a**) needlelike and (**b**) spherical.

Figure 2 shows the digital images of the porous Cu samples obtained using different shapes of carbamide particles with a volume content between 10 and 80%, where the upper shows needlelike porous Cu (NP-Cu), and the lower shows spherical porous Cu (SP-Cu). The average porosity of each porous Cu content is shown in Table 1. It can be seen that the porosity of the porous Cu samples increased with the increase in the spacer content. However, the effect of the shape of the space holder on the porosity did not show obvious regularity.

**Figure 3:**
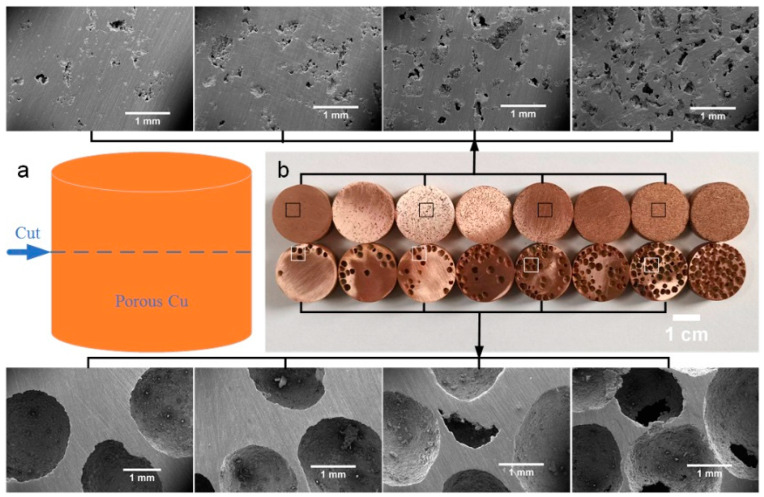
The digital and SEM images of the cross section of the porous Cu samples after being cut parallel to the outer surface at the middle part of the height with molybdenum wire: (**a**) diagram of the cutting site, (**b**) digital image of the cutting surface. The four SEM images above and below are the porous Cu samples prepared with 10%, 30%, 50%, and 70% spacer content.

Figure 3 shows the macro- and microtopography of the porous Cu samples in a cross-section. The cutting position diagram of the cross-section is shown in Figure 3a, and the actual cutting surface is shown in Figure 3b, where the upper layer is NP-Cu, and the lower layer is SP-Cu. As can be seen from the figure, the cross-section shows the holes that were not visible on the outer surface. These holes were generated from the removal of the space holder particles and are known as macropores. These macropores are also the main components of porous Cu’s porosity. Although the effect on the porosity was not regular, the shape of the space holder had a direct effect on the macropores. That is, the shape of the macropores maintained the shape of the space holder. Since the size of the needlelike space holder of carbamide was 180–380 μm, while the size of the SP-Cu was 0.85–3 mm, the size of the porosity in the case of the porous Cu using a needle-like space holder was smaller than that of SP-Cu.

### 3.2. Mechanical Properties

**Figure 4:**
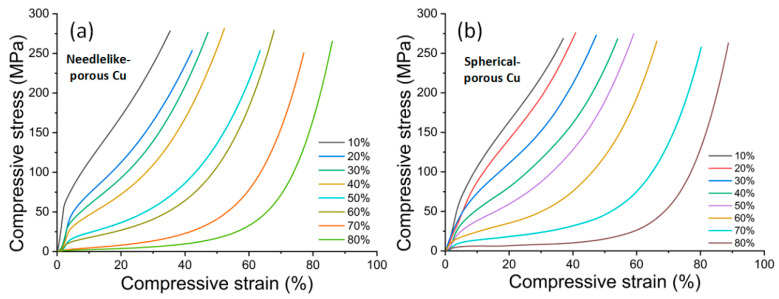
Compressive stress–strain curves of the samples using different shapes of space holders with a volume content between 10 and 80%: (**a**) NP-Cu and (**b**) SP-Cu.

Figure 4 shows the compressive stress–strain curves of NP-Cu and SP-Cu with different spacer contents. It can be seen that the stress–strain curve of the porous Cu sample was smooth, showing the deformation characteristics of plastic porous materials. The curve tended to be flat, and the stress level became more obvious with the increase in the spacer content (i.e., porosity). Meanwhile, the bends increased from one at low content to two at high content. Taking the highest content as an example, the curve transitioned from elasticity to plastic deformation at the first turn. At the second bend, the curve transitioned from plasticity to dense deformation. Obvious deformation zones were observed in the porous Cu with high spacer contents. One of the distinct differences between NP-Cu and SP-Cu was that the stress increased differently with the increase in the strain. Compared with the content, the shape of the space holder had less significant influence on the stress–strain curves.

**Figure 5:**
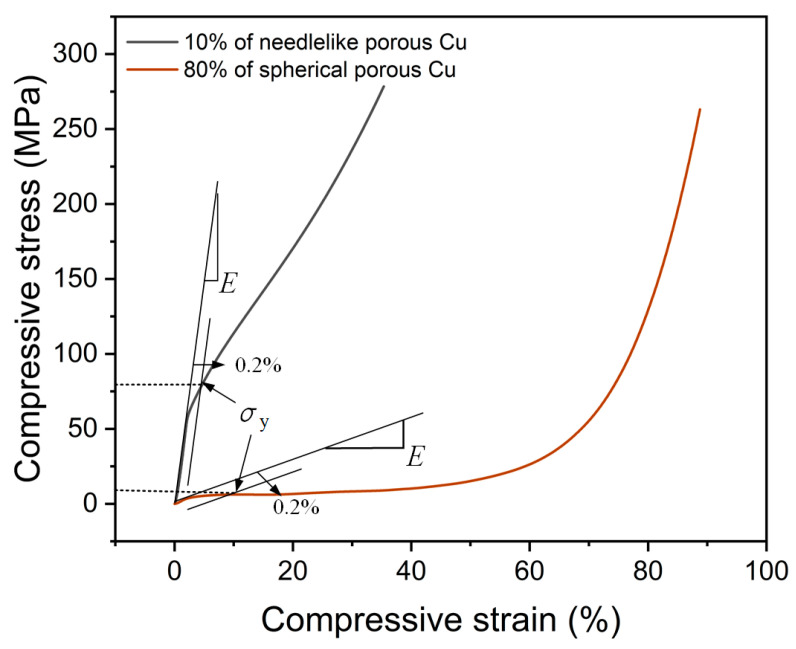
The calculation method of the elastic modulus and yield strength of the porous Cu samples.

The curves of all the samples showed that the stress increased monotonically with the increase in the strain; that is, there was no obvious yield point and no obvious stress peak. In this way, the elastic modulus was the proportional coefficient in the initial elastic deformation stage, and the yield strength was defined as the stress value of 0.2% residual deformation in the elastic limit [13], as shown in Figure 5, where *E* and *σ*_y_ represent the elastic modulus and yield strength of the porous Cu. These two curves were selected from the above figure for 10% NP-Cu and 80% SP-Cu.

The calculated elastic modulus and yield strength of the porous Cu samples are shown in Table 2. It can be seen that the elastic modulus and yield strength tended to decrease with the increase in the spacer content (i.e., porosity). As for the influence of the shape of the space holder, the elastic modulus of the NP-Cu was mostly higher than that of the SP-Cu with the same spacer content instead of 80%, while the yield strength of the SP-Cu was higher than that of the NP-Cu.

**Figure 6:**
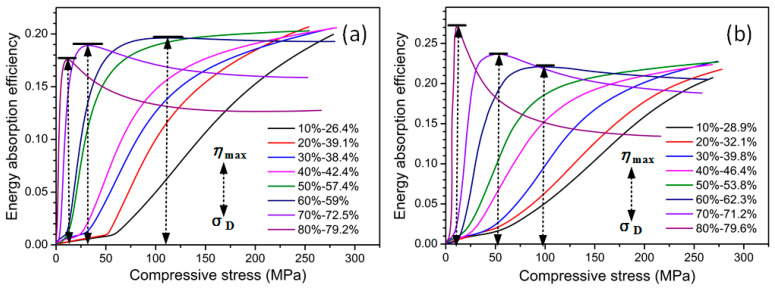
Energy absorption efficiency–compressive stress curves of porous Cu samples fabricated using different shapes of carbamide as the space holder with a volume content between 10 and 80%: (**a**) needlelike and (**b**) spherical. The black arrow indicates the maximum energy absorption efficiency at the upper part and the densification stress at the lower part.

Figure 6 shows the energy absorption efficiency–compressive stress curves of the porous Cu samples according to the above stress–strain curves. The energy absorption efficiency refers to the ratio of the absorbed energy to the corresponding stress, which was used to determine the best working state of the foam energy absorption [14]. The energy absorption efficiency increased monotonically with the increase in the stress when the spacer content was between 10 and 50%; however, when the spacer content was between 60 and 80%, the energy absorption efficiency first increased and then decreased with the increase in the stress (i.e., peak value). Therefore, the stress corresponding to the peak efficiency (*η*_max_) was the densification stress (*σ*_D_), and the strain corresponding to the intensity on the stress–strain curve was the densification strain (*ε*_D_). The densification stress is also called the compressive strength.

The calculated compressive strength and densification strain of the porous Cu samples with spacer contents between 60 and 80% are shown in Table 3. It can be seen that the compressive strength and densification strain decreased with the increase in the spacer content for the NP-Cu and SP-Cu. This is because the compressive strength decreases with the increase in the porosity, and the porosity increases with the increase in the space holder content; so, the compressive strength decreases with the increase in the space holder content. Due to the decrease in the compressive strength, the porous Cu sample entered the compaction stage earlier, although the stress platform tended to be flat with the increase in the space holder content. Compared with the NP-Cu, the compressive strength and densification strain of the SP-Cu were larger for the spacer contents of 60% and 70%, while they were smaller for the spacer content of 80%. Thus, the effect of the space holder shape on the compressive strength and densification strain of the porous Cu did not show a constant change rule.

In summary, the effect of the space holder shape on the mechanical properties of the porous Cu samples was not only significant but also regular when the spacer content was between 10 and 70%, ignoring 80%, although the effect of the porosity was not regular. In other words, the mechanical properties of the porous materials were not only affected by the porosity but also the pore shape. In fact, the change in the shape of the space holder also caused a change in the particle size. Therefore, different pore shapes and sizes were obtained with the same content, and the pore size also had an effect on the mechanical properties of porous Cu. Thus, to independently examine the effect of the shape of the space holder, one would need to ensure that the particle size was the same; however, this is very difficult to achieve. This may also explain why there are very few studies in the literature dealing with this area.

### 3.3. Performance Prediction

**Figure 7:**
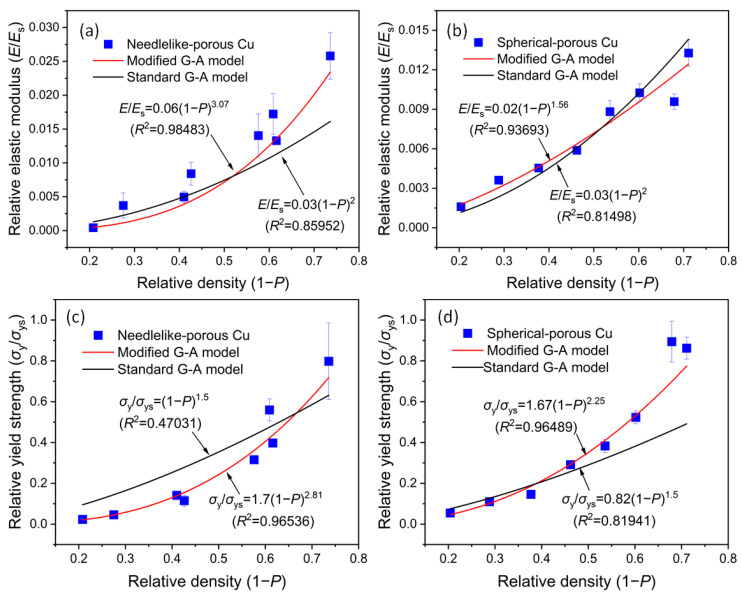
Plots of (**a**,**b**) the relative elastic modulus (*E*/*E*_s_) and the (**c**,**d**) relative yield strength (*σ_y_*/*σ_ys_*) versus the relative density (1 − *P*) with the experimentally obtained data, with standard and modified G-A model formulas for different kinds of porous Cu samples: (**a**,**c**) needlelike and (**b**,**d**) spherical. Here, *E*_s_ = 119 GPa, and *σ*_ys_ = 80 MPa, for the bulk pure Cu.

Although the shape of the space holder had no consistent effect on the porosity and mechanical properties of the porous Cu, we could still examine the effect of the shape of the space holders on the fitting equation through the relationship between them. Generally, the power index relation is widely used to describe the relationship between the elastic modulus and yield strength and the relative density of a porous metal or metal foams [10], which is also called the modified Gibson-Ashby (G-A) model. Figure 7 shows the relationships between the relative elastic modulus and yield strength and the relative density of the porous Cu samples based on the modified and standard G-A models, where *E*_s_ and *σ*_ys_ represent the elastic modulus and yield strength of the solid metal, respectively, and *E*/*E*_s_ and *σ*_y_/*σ*_ys_ represent the relative elastic modulus and yield strength, respectively. 1-*P* represents the relative density, *C*_1_ and *C*_2_ represent the coefficients, and *n*_1_ and *n*_2_ represent the exponents. *σ*_y_ is often replaced by the plateau stress (the flow stress of a plateau region in the compressive stress-strain curve) of a porous material [15]. The standard G-A model states that the relative elastic modulus of open porous metals is directly proportional to the square of the relative density, and the relative yield strength is directly proportional to the 1.5 square of the relative density [16,17].

It can be seen that the fitting degree of the red lines (i.e., modified G-A model) was significantly higher than that of the black lines (i.e., standard G-A model), which indicates that the modified model was more suitable for predicting the elastic modulus and yield strength of the present porous Cu than the standard G-A model. As far as the authors know, most of the previous studies have shown the same results (see Table 4).

According to Table 1, the relationship between the porosity (*P*) and spacer content (*x*) of the porous Cu samples was *P* = 0.73*x* + 0.19 (for NP-Cu) and *P* = 0.74*x* + 0.18 (for SP-Cu), respectively. By substituting the former into the formulas in Figure 7a,c and the latter into the formulas in Figure 7b,d, the relationships between the relative elastic modulus and yield strength and the spacer content of the porous Cu can be shown, according to the following formulas. Based on the above formulas, the prediction of the elastic modulus and yield strength of porous Cu, according to the content of the space holder, was realized.

(1) $$\frac{E}{E_{s}}=0.04{\left(0.81-0.73x\right)}^{3.07}\;(\mathrm{for}\;\mathrm{NP}-\mathrm{Cu})$$

(2) $$\frac{E}{E_{s}}=0.01{\left(0.82-0.74x\right)}^{1.56}\;(\mathrm{for}\;\mathrm{SP}-\mathrm{Cu})$$

(3) $$\frac{\sigma }{\sigma_{s}}=1.24{\left(0.81-0.73x\right)}^{2.81}\;(\mathrm{for}\;\mathrm{NP}-\mathrm{Cu})$$

(4) $$\frac{\sigma }{\sigma_{s}}=1.24{\left(0.82-0.74x\right)}^{2.25}\;(\mathrm{for}\;\mathrm{SP}-\mathrm{Cu})$$

## 4. Conclusions

The effects of the space holder shape on the pore structure and mechanical properties were studied herein. The porosity of the porous Cu samples increased with the increase in the spacer content; however, there was no regularity with the change in the space holder shape. Meanwhile, the pore shape of the porous Cu samples remained in the shape of space holder, and the pore connectivity increased with the increase in the porosity. Compared with the SP-Cu, it was easier for the NP-Cu to form an open structure due to the smaller size of the space holder used in the NP-Cu. The compressive stress–strain curves of the porous Cu samples were smooth, which showed the mechanical characteristics of porous plastic metals. The mechanical properties of the porous Cu samples decreased with the increase in the spacer content. Although the effect was not as significant as the content, the effect of the shape of the space holder on the mechanical properties of the porous Cu samples, which showed regularity, cannot be ignored; however, the effect of the shape of the space holder on the porosity of the porous Cu was not regular. The relationships between the relative elastic modulus and yield strength and the relative density were well described by a power law with an exponent of approximately 2–3. The results show that the space holder shape also had an influence on the index of fitting equation. Based on a dual model theory, the relationships between the above mechanical properties and spacer content were established. Thus, the source control of the mechanical properties of the porous Cu by the spacer content was realized.

## Figures and Tables

**Table 1:** Porosity of the obtained NP-Cu and SP-Cu samples in each group.

Spacer Content (%)	10	20	30	40	50	60	70	80
Porosity of NP-Cu/%	26.4 ± 1.5	39.1 ± 0.1	38.7 ± 0.8	42.4 ± 1	57.4 ± 0.5	59 ± 0.6	72.5 ± 0.1	79.3 ± 0.2
Porosity of SP-Cu/%	28.9 ± 1.1	32.1 ± 0.4	39.8 ± 0.9	46.4 ± 0.9	53.8 ± 0.9	62.3 ± 1	71.2 ± 0.1	79.7 ± 0.3

**Table 2:** Elastic modulus and yield strength of the porous Cu samples based on the above method.

Spacer Content (%)	Elastic Modulus (GPa)		Yield Strength (MPa)	
	NP-Cu	SP-Cu	NP-Cu	SP-Cu
10	3.07 ± 0.41	1.58 ± 0.12	63.85 ± 15.02	68.98 ± 4.29
20	2.05 ± 0.36	1.14 ± 0.07	44.78 ± 4.34	71.53 ± 7.99
30	1.58 ± 0.05	1.22 ± 0.08	31.82 ± 0.94	41.85 ± 2.54
40	1.67 ± 0.38	1.05 ± 0.10	25.26 ± 0.77	30.63 ± 2.62
50	1.00 ± 0.20	0.70 ± 0.03	9.21 ± 2.13	23.31 ± 1.49
60	0.59 ± 0.10	0.54 ± 0.03	11.29 ± 0.59	11.66 ± 0.97
70	0.44 ± 0.22	0.43 ± 0.02	3.67 ± 1.30	8.78 ± 0.76
80	0.05 ± 0.01	0.19 ± 0.02	1.85 ± 0.42	4.36 ± 0.32

**Table 3:** Compressive strength and densification strain of the porous Cu samples.

Spacer Content (%)	Compressive Strength (MPa)		Densification Strain (%)	
	NP-Cu	SP-Cu	NP-Cu	SP-Cu
60	117.65 ± 6.34	132.02 ± 35.99	51.35 ± 0.72	53.47 ± 3.57
70	33.26 ± 1.19	49.58 ± 8.15	45.78 ± 1.04	47.98 ± 1.74
80	12.07 ± 0.66	11.79 ± 2.45	45.43 ± 0.45	43.47 ± 5.08

**Table 4:** The relations between the mechanical properties and the porosity of porous metals in the literature.

Literature	Skeleton Metal	Elastic Modulus	Yield Strength	Porosity (%)
Ray [5]	Cu	*E*/*E*_s_ = (1 − *P*)^(2.6−4.14)^	-	38.5–70.4
Hong [18]	Cu	-	*σ*_y_/*σ*_ys_ = 0.6(1 − *P*)^2.28^	70.2–76.6
Jana [6]	Cu	*E*/*E*_s_ = 192.61 − 11.09*P* + 0.27*P*^2^ − 0.003*P*^3^ + 1.39 × 10^−5^*P*^4^		27.5–67.1
Badwe [19]	Au	*E*/*E*_s_ = 0.86(1 − *P*)^2.8^	-	43–70
Bolzoni [20]	Ti	*E*/*E*_s_ = 0.79(1 − *P*)^2.49^	-	44.3–71.1
	Fe	*E*/*E*_s_ = 0.90(1 − *P*)^2.54^		38.1–75.6
Jenei [21]	Ti	*E*/*E*_s_ = 1.3(1 − *P*)^4.6^	*σ*_y_/*σ*_ys_ = 10.2(1 − *P*)^5^	51–68.4
Tuncer [22]	Ti	-	*σ*_y_/*σ*_ys_ = 1.07(1 − *P*)^2.28−2.57^	44–68
Cheneler [23]	Al	*E*/*E*_s_ = 7.38(1 − *P*)^2.71^	*σ*_y_/*σ*_ys_ = 24.6(1 − *P*)^2.71^	~80–85%
		*E*/*E*_s_ = 5.32(1 − *P*)^2.92^	*σ*_y_/*σ*_ys_ = 21.81(1 − *P*)^3.09^	

## Data Availability

The original contributions presented in the study are included in the article, further inquiries can be directed to the corresponding authors.
